# Supplementary material for: Recommendations to Improve Tick-Borne Encephalitis Surveillance and Vaccine Uptake in Europe
Source: Microorganisms. 2022 Jun 24;10(7):1283. doi: 10.3390/microorganisms10071283 (PMC9322045; doi:10.3390/microorganisms10071283)
Supplement: Supplementary file 1 [file microorganisms-10-01283-s001.zip › microorganisms-1757983-supplementary.pdf]

microorganisms

MDPI

Supplementary Table S1. European national TBE diagnostic criteria.

**Country**	**TBE diagnostic criteria**
Austria<br>Belgium<br>Bulgaria<br>Croatia<br>Czech Republic<br>Estonia<br>Finland<br>France<br>Hungary<br>Ireland<br>Latvia<br>Netherlands<br>Norway<br>Poland<br>Romania<br>Slovakia<br>Spain<br>Sweden<br>United Kingdom	EU-2012/2018 [14,38]<br>**Clinical Criteria**<br>Any person with symptoms of inflammation of the CNS (for example, meningitis, meningo-encephalitis, encephalomyelitis, encephaloradiculitis)<br><br>**Laboratory Criteria**<br>Laboratory criteria for case confirmation:<br>At least one of the following five:<br>• TBE specific IgM AND IgG antibodies in blood<br>• TBE specific IgM antibodies in CSF<br>• Seroconversion or four-fold increase of TBE-specific antibodies in paired serum samples<br>• Detection of TBE viral nucleic acid in a clinical specimen,<br>• Isolation of TBE virus from clinical specimen<br>Laboratory criteria for a probable case:<br>• Detection of TBE-specific IgM-antibodies in a unique serum sample<br>• Epidemiological Criteria<br>• Exposure to a common source (unpasteurized dairy products)<br>Case Classification<br>A. Possible case NA<br>B. Probable case<br>Any person meeting the clinical criteria and the laboratory criteria for a probable case,<br>OR<br>Any person meeting the clinical criteria with an epidemiological link<br>C. Confirmed case<br>Any person meeting the clinical and laboratory criteria for case confirmation<br><br>Note: Serological results should be interpreted according to previous exposure to other flaviviral infections and the flavivirus vaccination status. Confirmed cases in such situations should be validated by serum neutralization assay or other equivalent assays.
Slovenia	Confirmed TBE case:<br>• Clinical signs/symptoms of meningitis or meningoencephalitis, and<br>• Elevated CSF cell count (> 5 x 10^6 leukocytes/L), and<br>• Demonstration of recent infection with TBEV:<br>• Presence of serum IgM and IgG antibodies to TBEV<br>• In patients previously vaccinated against TBE (or exposure to other flaviviral infections and the flavivirus vaccination) demonstration of intrathecal synthesis of IgG antibodies to TBEV
Germany [40]	Either non-specific symptoms (at least 2 of the following: Chills, severe malaise, headache and muscle, limb or back pain) or signs of central nervous system (CNS) infection (meningitis, encephalitis or myelitis separately or in combination))<br>AND<br>Laboratory confirmation by means of either simultaneously elevated IgM and IgG TBE-specific antibodies in serum or cerebrospinal fluid (CSF) or an increase in TBE-specific IgG antibodies in serum or the detection of intrathecal antibody synthesis]
Italy [14]	Clinical criteria: any symptoms of inflammation of the CNS (for example, meningitis, meningo-encephalitis, encephalomyelitis, encephaloradiculitis).<br>Confirmed case: At least one of the following five laboratory criteria:<br>• TBE specific IgM and IgG antibodies in blood;

Microorganisms **2022**, *10*, 1283. https://doi.org/10.3390/microorganisms10071283 www.mdpi.com/journal/microorganisms

	• TBE specific IgM antibodies in CSF;<br>• seroconversion or four-fold increase of TBE-specific antibodies in paired serum samples; detection of TBE viral nucleic acid in a clinical specimen;<br>• isolation of TBE virus from clinical specimen.
Luxembourg	Not specified/unknown
Serbia	No national diagnostic criteria.<br>Since January 2020, surveillance according to the EU Clinical Case Definition is introduced in all hospitals in Autonomous Province of Vojvodina, as a part of Special Public Health Program.
Ukraine [43]	Probable case:<br>— Clinical: fever > 38.5°C and neurological signs.<br>— Laboratory diagnosis: detection of specific IgM.<br>Confirmed case:<br>— Clinical: signs of CNS involvement.<br>— Laboratory diagnosis:<br>— Detection of specific IgM or/and IgG in serum or CSR;<br>— Or 4-fold increase specific IgG (interval between first and second serum of at least 2 weeks);<br>— Or PCR positive test.<br>— Epidemiological: exposure to tick bite or unpasteurized dairy products
